# Hereditary angioedema: A rare but serious and commonly misdiagnosed disease

**DOI:** 10.1097/01.NURSE.0000891944.11247.83

**Published:** 2022-11-17

**Authors:** Lisa Zacek

**Affiliations:** **Lisa Zacek** is an infusion RN at the Midwest Immunology Clinic in Plymouth, Minn.

**Keywords:** angioedema, C1 inhibitor, hereditary angioedema

## Abstract

Hereditary angioedema is a rare and commonly misdiagnosed disease characterized by recurrent, painful, nonurticarial, and nonpruritic deep tissue swelling attacks, including potentially life-threatening asphyxiation. Nurses can assist in identifying disease hallmarks and provide emergency care, patient support, and education about injectable or infused medications.

## Abstract 2

Hereditary angioedema is a rare and commonly misdiagnosed disease. This article discusses how nurses can assist in identifying disease hallmarks and provide emergency care, patient support, and education.

## Clinical vignette

**Figure:**
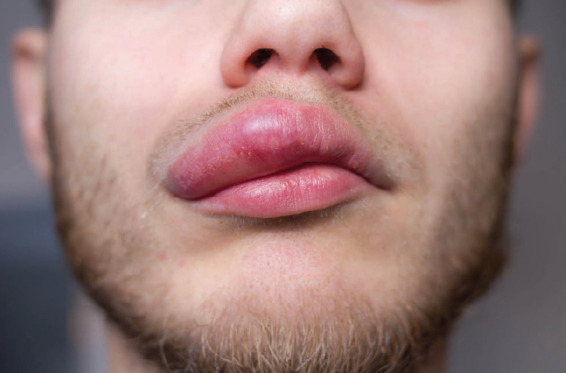
No caption available.

RJ, a 13-year-old high school student presents to her primary care provider complaining of very swollen hands and forearms, which have worsened over the past 18 hours to the point where she could not go to school. She has no history of food or contact allergies and is taking no medications. Her mother had given her 50 mg of diphenhydramine when she woke up, but this did not confer any apparent improvement. The patient believes her face was starting to swell as well, but there was no evidence of hoarseness or trouble breathing. She denies pruritus and there is no evidence of urticaria (also called wheals).

A thorough history revealed that a similar, albeit less severe, event had occurred about a year prior soon after the onset of menses. RJ also admitted to occasional episodes of gastrointestinal pain that each lasted for a few days with no determined cause and resolved without treatment. Based on this history, which seemed indicative of hereditary angioedema, and the risks of asphyxiation with laryngeal swelling, she was sent to a nearby ED where plasma-derived C1 esterase inhibitor (pdC1-INH) was administered I.V. and RJ was monitored for several hours in the ED until the facial and peripheral swelling resolved. Follow-up lab testing revealed low levels of complement component 4 (C4), C1-INH antigen, and C1-INH function, confirming a diagnosis of type 1 hereditary angioedema (HAE).

## Hereditary angioedema

HAE is an autosomal dominant disease characterized by recurrent, generally unpredictable episodes of localized angioedema.^2^ The angioedema attacks can take days to subside if untreated and have the potential to be life-threatening if they affect the larynx. HAE is rare, with an estimated prevalence of approximately 1 in 50,000 people, which translates to roughly 6,000 patients with HAE in the US.^1^-^3^ Patients with HAE are managed differently than patients with other forms of angioedema and will not respond to typical interventions such as epinephrine and high-dose antihistamines. For these reasons, it is critical to understand the presentation and acute treatment of patients with HAE. Many tell-tale indicators of HAE can be teased out by patient history and careful review of attack characteristics, even during a telemedicine visit.

## Pathophysiology

HAE attacks result from localized production of excess bradykinin, a potent vasodilatory mediator with vascular permeability-enhancing effects, following activation of the plasma kallikrein-kinin system, which is normally regulated by the C1-INH enzyme. Most patients with HAE have genetic mutations resulting in either deficient levels of normally functioning C1-INH (HAE-1; 85% of HAE cases with functional C1-INH deficiency) or C1-INH proteins that are dysfunctional, even though levels may be normal (HAE-2; ~15% of cases with functional C1-INH deficiency).^4^ A third type of HAE has recently been identified in which C1-INH levels and function are normal (HAE-nC1-INH)^1^; these cases seem to be related to a variety of different genetic mutations.^4^,^5^ The following information pertains primarily to HAE-1 and HAE-2.

**Figure:**
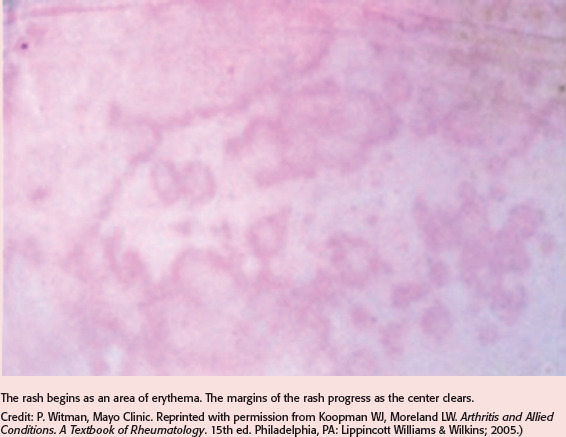
Erythema marginatum

## Clinical manifestations

Patients often report experiencing a “prodrome” or aura shortly before the onset of an HAE attack. The most common prodrome event is erythema marginatum, a distinctive, pale pink rash caused by injured or inflamed capillaries that spreads over the limbs or torso (see *Erythema marginatum*). Other reported prodrome symptoms include tingling, discomfort, fatigue, weakness, or lack of energy.^2^

Angioedema attacks, which can be painful and disfiguring, typically affect subcutaneous tissues of the extremities (arms or legs, hands or feet) or the face and lips (see *Angioedema of lip*). They can also occur in the submucosal tissues of the oropharynx and larynx, or abdomen, and occasionally, in the genitals.^2^,^6^ Abdominal attacks are the most common and are generally associated with intense abdominal distension and pain, nausea, vomiting, diarrhea, and hypotension.^7^ Laryngeal attacks are less common overall, but roughly half of the patients with HAE experience at least one such attack in their lifetime.^8^ Laryngeal attacks can result in death due to asphyxiation or permanent disability from hypoxic brain damage if not treated appropriately and quickly.^9^ HAE-associated angioedema can start in one location and progress to another.^10^ All HAE attacks carry the potential to progress to laryngeal swelling and fatal asphyxiation.^11^ Mortality by asphyxiation associated with an untreated laryngeal HAE attack is about 30%.^4^ Unlike more common forms of angioedema, HAE-related swelling is not accompanied by urticaria or pruritus. Urticaria and pruritus are more commonly observed with histamine-mediated angioedema, which is triggered by mast cell degranulation and histamine release.^4^

**Figure:**
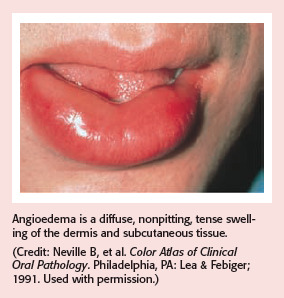
Angioedema of lip

## HAE triggers

Most HAE attacks occur spontaneously with no apparent triggering event. However, attacks can be triggered by factors such as stressful life events, invasive medical or dental procedures, physical trauma, recent infection, and fatigue.^2^ Because estrogen plays a role in controlling bradykinin production, the clinical course of HAE varies during the hormonal stages of a woman's life, from puberty to monthly menses, pregnancy, and menopause.^3^,^12^ Medications can exacerbate the frequency and severity of HAE attacks including estrogen-containing medications and angiotensin-converting enzyme inhibitors.

**Table:** Recommended therapies for HAE^1^,^3^

	WAO/EAACI (2021)	US HAEA (2020)
*On-demand treatment* First-line Second-line Third-line	C1-INH,^a^ ecallantide,^b^ or icatibant Solvent detergent-treated plasma Fresh frozen plasma	C1-INH,^a^ ecallantide,^b^ or icatibant Solvent detergent-treated plasma Fresh frozen plasma
*Short-term prophylaxis* First-line Second-lineThird-line	I.V. plasma-derived C1-INH Fresh frozen plasma Attenuated androgens^c^	Plasma-derived C1-INH Anabolic androgen^c^ Fresh frozen plasma
*Long-term prophylaxis* First-line Second-line	Plasma-derived C1-INH, lanadelumab, berotralstat Attenuated androgens	Plasma-derived C1-INH, lanadelumab Anabolic androgens, antifibrinolytics
*FDA-approved after guideline development*		Berotralstat

a Plasma-derived or recombinant.

b Administered by a healthcare professional with appropriate medical support to manage anaphylaxis.

c Contraindicated in children, pregnant, and nursing women.

C1-INH = C1 inhibitor; I.V., intravenous; US HAEA = United States Hereditary Angioedema Association guidelines; WAO/EAACI = World Allergy Organization/European Academy of Allergy and Clinical Immunology guidelines

## Diagnosis

Episodes of unpredictable angioedema that are not accompanied by urticaria and pruritus should raise clinical suspicion of HAE. The onset of signs and symptoms in childhood (mean age of onset, 8 to 12 years) and a family history of recurrent angioedema are other indicators suggestive of HAE.^1^,^2^

Confirmation of an HAE diagnosis and determination of HAE type may be obtained by lab evaluation of plasma levels and function of C1-INH and C4, which is a complement protein that is consumed upon uncontrolled activation of the complement pathway in the absence of normal C1-INH function.^6^

A low C4 level is highly suggestive of either HAE-1 or HAE-2. A C4 level can be used as an initial screening test for HAE. HAE can be definitively diagnosed by an abnormally low level of C1-INH function even if levels are normal.^1^-^3^ C1-INH antigenic levels, such as a reduced C1-INH quantity, are low in HAE-1 and usually normal in HAE-2. Conversely, HAE-2 usually returns normal antigenic levels (quantity of C1-INH); however, C1-INH function is low because the protein is abnormal in HAE-2.^6^ For clinical purposes, these two types of HAE are managed in the same way.^3^

## Management

Because HAE attacks are mediated by excess bradykinin and are not related to mast cell activation and histamine release, they require HAE-specific therapy and do not respond to treatment with antihistamines, epinephrine, or corticosteroids.^2^,^3^ This critical difference is the most pressing reason for the need to accurately diagnose angioedema type, especially given the potentially fatal nature of HAE attacks. First-line therapies for HAE act by replacing the C1-INH that is deficient or dysfunctional or by inhibiting the production or function of bradykinin.

**Table:** FDA-approved medications for on-demand treatment of HAE attacks^a^,^3^,^10^,^13^-^17^

	Plasma-derived C1-INH	Icatibant	Ecallantide	Recombinant human C1-INH
US indication	Children, adults	Adults at least 18 years old	12 years of age and older	Adolescents, adults
Route of administration	I.V.	SUBQ	SUBQ	I.V.
Self-administration	Yes	Yes	No^b^	Yes
MOA	Replaces missing or malfunctioning C1-INH^c^	Inhibits bradykinin from binding the B2 receptor	Inhibits plasma kallikrein	Increases plasma levels of functional C1-INH activity^c^
Potential AE	Anaphylaxis (rare); transmission of infectious agent (theoretical)	Injection site reactions (common)	Anaphylaxis (2.7%)^17^	Anaphylaxis (uncommon)

a Data in this table are not derived from head-to-head studies.

b Ecallantide should only be administered by a healthcare professional with appropriate medical support to manage anaphylaxis and HAE.

c Inhibits plasma kallikrein and factors in the coagulation, complement, and fibrinolytic pathways.

AE=adverse events; C1INH=C1 inhibitor; I.V.=intravenous; HAE=hereditary angioedema; MOA=mechanism of action; SUBQ=subcutaneous

There are three main HAE management strategies (see *Recommended therapies for HAE*):

### Treatment of acute attacks (“on-demand” treatment)

Management of HAE attacks requires promptly administered on-demand therapies to limit attack progression and severity 3 The FDA-approved on-demand treatments for HAE include:

- I.V. Human plasma-derived C1-INH concentrate (pdC1-INH)
- Recombinant human C1-INH (rhC1-INH)
- Icatibant, a bradykinin B_2_-receptor antagonist
- Ecallantide, a kallikrein inhibitor (available only in the US; see *FDA-approved medications for on-demand treatment of HAE attacks*).

The first three therapies can be self-administered by the patient or a caregiver; ecallantide requires administration by a healthcare professional with appropriate medical support to manage anaphylaxis, which occurs in about 4% of patients treated with ecallantide. Self-administration of on-demand treatment allows patients to limit the morbidity and potential mortality of an attack by initiating treatment as soon as possible. The onset of action following early treatment is usually within 30 to 120 minutes.^3^ Guidelines from the World Allergy Organization/European Academy of Allergy and Clinical Immunology (WAO/EAACI) recommend that all patients with HAE maintain a supply of medication sufficient for on-demand treatment of two attacks and that they carry on-demand treatment with them at all times.^1^

### Long-term prophylaxis (LTP)

When deciding to place a patient with HAE on LTP, the healthcare provider (HCP) should consider factors, such as the level of disease activity, frequency of attacks, the patient's quality of life, availability of healthcare resources, and failure to achieve adequate control with appropriate on-demand therapy.^1^,^3^ Eliminating or greatly reducing the frequency of HAE with effective LTP gives patients greater control and quality of life.

The most recently updated HAE guidelines (United States Hereditary Angioedema Association [US HAEA] and the WAO/EAACI) recommend either I.V. or SUBQ pdC1-INH, or the SUBQ plasma kallikrein inhibitor (monoclonal antibody) lanadelumab-flyo, as preferred first-line agents for LTP (see *FDA-approved medications for LTP of HAE attacks*).^1^,^3^ An oral kallikrein inhibitor, berotralstat, is recommended as a first-line option for LTP by the WAO/EAACI 2021 guidelines; it was granted FDA approval for LTP after the most recent US HAEA guideline updates were published and is not yet reflected in the prioritization of LTP therapies in that document.

### Short-term prophylaxis

Short-term, or preprocedural, prophylaxis (STP) is strongly recommended whenever a patient with HAE is preparing to undergo an invasive dental or medical procedure or is facing a stressful life event. Short-term prophylaxis can be a single dose of I.V. pdC1-INH 1-12 hours before the stressor, or anabolic androgen started 5 to 7 days before the stressor and continued for 2 to 5 days afterward. In a large, retrospective study, facial swelling or laryngeal attacks occurred in 20.8% of patients who received STP with I.V. pdC1-INH 1 hour before a dental extraction; in comparison, 37.2% of patients who did not receive STP experienced facial or laryngeal edema. On a per-patient basis, STP led to a 44.1% reduction in angioedema attacks.^22^

**Table:** FDA-approved medications for LTP of HAE attacks^a^,^1^,^3^,^10^,^18^-^21^

	Plasma-derived C1-INH	Plasma-derived C1-INH	Lanadelumab-flyo	Berotralstat	Danazol
US indication	Adults, adolescents, and pediatric patients (6 years of age and older)	6 years of age and older	Adult and pediatric patients 12 years and older	Adults and pediatric patients 12 years and older	Adults under 65
Route of administration	I.V.	SUBQ	SUBQ	Oral	Oral
Self-administration	Yes	Yes	Yes	Yes	Yes
MOA	Increases plasma levels of C1-INH activity^b^	Replaces the missing or malfunctioning C1-INH^b^	Inhibits plasma kallikrein	Decreases plasma kallikrein activity to control excess bradykinin generation	Increases deficient C1-INH and C4 levels
Potential AEs	Risk of anaphylaxis (rare); transmission of infectious agent (theoretical)	Risk of anaphylaxis (rare); transmission of infectious agent (theoretical)	Risk of anaphylaxis (rare); injection site reactions (common)	Abdominal pain, vomiting, diarrhea, back pain, gastroesophageal reflux disease	Weight gain, virilization, acne, altered libido, muscle pains/cramps, headaches, depression, fatigue, nausea, constipation, menstrual abnormalities, increase in liver enzymes, hypertension, and alterations in lipid profile (common)

a Data in this table are not derived from head-to-head studies.

b Inhibits plasma kallikrein and factors in the coagulation, complement, and fibrinolytic pathways.

AE = adverse event; C1-INH = C1 inhibitor; I.V. = intravenous; HAE = hereditary angioedema; MOA = mechanism of action; SUBQ = cutaneous

C1-INH products are recommended as first-line options for STP; a course of oral anabolic androgens is a second-line option. If none of these agents are available or there is no time for a course of anabolic androgen, fresh frozen plasma may be used as it contains C1-INH.^1^,^3^ There is always the risk of a breakthrough attack with STP, so patients with HAE should remain under observation after invasive dental or medical procedures with rapid access to the patient's usual on-demand therapy.^1^

## Management in special populations

### Pregnant and lactating women

While there are no HAE treatments that are FDA-approved for use during pregnancy, the 2020 US HAEA guidelines recommend C1-INH for attack treatment in women who are pregnant or breastfeeding, a recommendation based on case reports and observational study data; prophylaxis is recommended for pregnant women before medical procedures, such as amniocentesis, chorionic villus sampling, and also before planned cesarean delivery and deliveries requiring vacuum or forceps. Attacks are uncommon during normal vaginal deliveries, but on-demand treatment should be available as a precaution. The guidelines also recommend C1-INH products for on-demand treatment or prophylaxis in women who are breastfeeding. Anabolic androgens are contraindicated in those who are pregnant or lactating.^3^

### Pediatrics

HAE commonly manifests during childhood. The mean age of onset is 10 years, and signs and symptoms usually worsen around the time of puberty.^23^ Several HAE medications have been FDA-approved for use in pediatric patients, including pdC1-INH (approved for treatment in “children” and prophylaxis in patients at least 6 years of age), ecallantide (approved for treatment in patients at least 12 years of age), recombinant C1-INH (approved for prophylaxis in “adolescents”), and lanadelumab and berotralstat (both approved for prophylaxis in patients at least 12 years of age). The most recent update of the WAO/EAACI guidelines recommends the use of C1-INH or icatibant to treat attacks in patients younger than 12 years of age, and also recommends pdC1-INH (not recombinant C1-INH) as the preferred choice for LTP in children younger than 12.^1^ According to the 2020 US HAEA guidelines, the preferred form of LTP for children is SUBQ pdC1-INH due to the greater tolerability of repeated subcutaneous access versus repeated I.V. access (FDA-approved for children over 12 years of age). Anabolic androgens are contraindicated in children.^3^

## Nursing considerations

HAE is not a common diagnosis, yet one that requires awareness and vigilance. The unique pathophysiology and treatment requirements of HAE demand accurate diagnosis to avoid potentially life-threatening consequences and improve the patient's quality of life.

Low initial suspicion has been deemed the most significant hurdle to the diagnosis of HAE in primary care clinics and EDs.^24^ Nurses are often the first point of patient contact; asking appropriate questions can assist in identifying any disease hallmarks. Outside of specialized professions, such as allergy and immunology, it cannot be assumed that all HCPs are aware of HAE as a potential diagnosis. A suspicion of HAE should be raised when patients present with nonurticarial, nonpruritic swelling that has no apparent cause, such as no known food or contact allergies, and a previous lack of response to typical treatments such as steroids and antihistamines, and also when patients describe a personal or family history of recurrent episodes of unexplained abdominal pain. A few simple blood tests, such as C4, C1-INH antigen, and C1-INH function, can confirm a diagnosis of HAE.

The awareness of potentially fatal laryngeal swelling is also very important. Patients who present with any facial or laryngeal swelling should be managed as an emergency. If they have any swelling above the shoulders, patients can be counseled to administer on-demand treatment and then go to a local ED or call 911 in case of the rare event that the swelling progresses into potentially fatal laryngeal edema, which can happen relatively quickly (within hours).

Finally, almost all of the currently approved medications for HAE are injectable, either I.V. or SUBQ. Nurses can play a valuable role in teaching patients to self-administer these medications and offering regular follow-up appointments. Follow-up frequency, for new patients, is about every 3 months to monitor attack frequency and make any necessary adjustments to the treatment regimen. Once stabilized on a given treatment regimen, patients are generally seen every 6 to 12 months for routine follow-up.

The potential adverse reactions of each prescribed medication should be reviewed with the patient. They should be educated on how to differentiate between the adverse reactions of drugs and HAE signs and symptoms. Patient information material, part of drug labeling supplied as a paper copy within the drug packaging and also available online, can be a helpful resource for patients. While HAE attacks are often spontaneous, patients should be made aware of the potential for attacks to be precipitated by dental work, invasive medical procedures, or stressful events. Female patients should be alerted to the potential effects of estrogen-containing medications such as oral contraceptives on HAE attack frequency.

The information presented here is far from an exhaustive review of HAE and its treatments. Further, it should be noted that several non-HAE types of angioedema are also mediated by excess bradykinin production, including angioedema induced by angiotensin-converting enzyme-inhibitor therapy in susceptible individuals, and acquired angioedema that may be associated with an autoimmune mechanism. Nurses should consult more detailed HAE and HAE management guidelines and other resources to assist with patient care if referral to an HAE specialist is not an option (see *HAE resources*).^1^-^3^ For example, the organization HAE International provides downloadable HAE emergency cards in multiple languages that patients can use to share relevant HAE-related information in the event of an emergency, and also a patient newsletter. Many of the HAE drug manufacturers also offer a variety of patient resources online. The morbidity and mortality of HAE can be greatly lessened by a timely and accurate diagnosis, implementation of appropriate therapy, and referral to—or treatment guidance from—an HAE expert.

## HAE resources

***US Hereditary Angioedema Association***

www.haea.org

(866) 798-5598

***Hereditary Angioedema International (HAEi)***

www.haei.org

***American Academy of Asthma and Immunology***

www.aaaai.org
